# Individual differences in white matter microstructure of the face processing brain network are more differentiated from global fibers with increasing ability

**DOI:** 10.1038/s41598-022-17850-4

**Published:** 2022-08-18

**Authors:** Xinyang Liu, Mattis Geiger, Changsong Zhou, Andrea Hildebrandt

**Affiliations:** 1 Shanghai Key Laboratory of Brain Functional Genomics (Ministry of Education), School of Psychology and Cognitive Science, East China Normal University, Shanghai, 200062 China; 2 Department of Psychology, Carl von Ossietzky University of Oldenburg, 26129 Oldenburg, Germany; 3 Department of Physics, Centre for Nonlinear Studies, Institute of Computational and Theoretical Studies, Hong Kong Baptist University, Kowloon Tong, Hong Kong; 4 Institute of Psychology and Education, University of Ulm, 89069 Ulm, Germany; 5 Department of Implementation Research, Bernhard Nocht Institute for Tropical Medicine, 20359 Hamburg, Germany; 6 Research Center Neurosensory Science, Carl von Ossietzky University of Oldenburg, 26129 Oldenburg, Germany

**Keywords:** Cognitive neuroscience, Social behaviour, Social neuroscience

## Abstract

Face processing—a crucial social ability—is known to be carried out in multiple dedicated brain regions which form a distinguishable network. Previous studies on face processing mainly targeted the functionality of face-selective grey matter regions. Thus, it is still partly unknown how white matter structures within the face network underpins abilities in this domain. Furthermore, how relevant abilities modulate the relationship between face-selective and global fibers remains to be discovered. Here, we aimed to fill these gaps by exploring linear and non-linear associations between microstructural properties of brain fibers (namely fractional anisotropy, mean diffusivity, axial and radial diffusivity) and face processing ability. Using structural equation modeling, we found significant linear associations between specific properties of fibers in the face network and face processing ability in a young adult sample (*N* = 1025) of the Human Connectome Project. Furthermore, individual differences in the microstructural properties of the face processing brain system tended toward stronger differentiation from global brain fibers with increasing ability. This is especially the case in the low or high ability range. Overall, our study provides novel evidence for ability-dependent specialization of brain structure in the face network, which promotes a comprehensive understanding of face selectivity.

## Introduction

Face processing is a crucial social ability demonstrated to be distinct from general cognition^1–5^. In line with elevated functional activation to faces as compared with other categories of complex object stimuli, a cluster of brain regions was concluded to be face-selective^6,7^, presently referred to as the face processing brain network^8–10^. In a considerable number of previous studies on the neural structure and mechanisms underlying face processing, a stronger emphasis was set on investigating grey matter regions and functional connectivity. White matter connections were somewhat neglected^11,12^. Thus, little is known on how white matter connectivity within the face processing brain network is related with performance in the face processing domain. Furthermore, it is unacquainted whether the white matter structure of the face network is more specialized with higher face or general cognition, especially in young adulthood, the developmental peak of most abilities.

To capture properties of the brain white matter tracts in vivo, a brain imaging technique known as diffusion weighted imaging (DWI) has been extensively used in recent years^13^. To quantify the microstructural properties of the fibers, fractional anisotropy (FA), mean diffusivity (MD), axial diffusivity (AD) and radial diffusivity (RD) are among the most widely used parameters^14,15^. Larger FA values indicate stronger overall diffusion directionality of water molecules in brain regions, which can be modulated by mechanisms such as enhanced fiber myelination, lower axon diameter, higher fiber density and so on^14^. MD describes the average of diffusion in all directions, which can be a non-specific but sensitive measure to reflect variations of brain tissue^14^. AD and RD respectively indicate the diffusion intensity along and perpendicular to the principal diffusion direction^15^.

White matter connections have been detected by fiber tracing approaches within the functional face processing brain system before^16,17^. Generally, the fusiform face area (FFA), the occipital face area (OFA) and the posterior superior temporal sulcus (pSTS) are considered to form the core face processing system, responsible for early perception of invariant facial features and of the dynamic visual appearance of faces^6,7^. Further areas, such as the anterior temporal lobe (ATL) and the intraparietal sulcus (IPS) are referred to as the extended face system, dedicated to process higher level information such as facial identity, facial expressions, biographical information about a person and the like. In a recent study, Liu and colleagues^9^ discovered that individual differences in the FA of brain fibers connecting functional ROIs in the face network (referred to as functionally defined fibers) can be explained by two independent factors corresponding to what are known as functional core versus extended face processing brain systems^6,7^.

Brain white matter tracts grant a solid structural foundation for functional brain interactions which subserve various cognitive abilities^18^. In line with this assumption, previous studies demonstrated linear relationships between white matter microstructural properties and cognitive abilities, including the face processing domain. Several global brain fibers, such as the inferior longitudinal fasciculus (ILF), the inferior fronto-occipital fasciculus (IFOF) and the superior longitudinal fasciculus (SLF) were shown to be associated with face processing performance^11,12^. In one study applying a more specific fiber selection in young and middle-aged adults, a significant relationship was reported between the FA of a right hemisphere fiber passing through the mid-fusiform face-selective brain region (FFA2) and face processing accuracy measured with one behavioral task^19^. Besides, the MD values of this fiber at both hemispheres were significantly lower among participants with developmental prosopagnosia as compared with healthy adults.

Aiming to better understand how the brain supports complex cognition, neural differentiation depending on the level of ability was also targeted in previous studies. Ability dependent differentiation and dedifferentiation of cognitive functions is an influential hypothesis derived from Spearman’s law of diminishing returns. It postulates at higher ability levels, cognitive domains are more differentiated^20^. Furthermore, neuroimaging techniques were applied to understand the neural mechanisms underlying the observed differentiation and dedifferentiation of cognitive abilities across age and ability levels^21–23^. With respect to face processing, fMRI studies revealed age-related dedifferentiation in both core and extended systems accompanied by attenuated activity in face selective brain regions^24,25^. Besides, broadened involvement of compensational processing areas (e.g., the frontal lobe) were also found in elderly suffering from cognitive ability loss^24,25^. In another study, modulation of face task demands on functional connectivity strength within the core face network was found in young adults but not among children^26^, demonstrating differentiation across early development.

All the above studies together suggest a strong functional modulation and differentiation of the face processing brain system among young adults during the peak of developmental trajectories of cognitive abilities as compared to elderly and children^27–29^. Note that ability- and age-related differentiation-dedifferentiation were often confounded in previous studies. To our knowledge, no study investigated the neuroanatomical underpinnings of ability-related differentiation of face processing in an age homogenous sample, which allows to purely target ability-differentiation because age-related variation is eliminated. Previous findings have set a solid foundation demonstrating correspondence between brain structure and function in face processing^9,11,19^. Therefore, brain functional differentiation-dedifferentiation in this cognitive domain could probably be further extended to the investigation of brain structure. This will help to achieve a comprehensive understanding of the face selectivity phenomenon at the neural and behavioral levels.

This study is pursuing two goals. (1) It aims to investigate whether individual differences in microstructural properties of the fibers belonging to the face processing brain network are linearly associated with face processing speed and accuracy in younger age. These associations will be tested incrementally above those between global brain fibers and general cognitive abilities. (2) We study whether individuals with higher face and general processing ability are characterized by a more differentiated structural face processing brain network as compared to global brain fibers. Such a finding would be in line with the idea that higher cognitive performance goes along with a more specialized brain system as compared with global microstructural properties of the brain.

## Results

### On the linear relationship between white matter microstructural factors and performance speed

Linear brain-behavior structural models were estimated to investigate whether individual differences in white matter microstructure of the face processing brain network, indicated by tract-averaged diffusion MRI metrics namely FA, MD, AD and RD, are associated with processing speed and response accuracy in face processing. Associations were simultaneously modeled and compared with those of global brain fiber tract structure and general cognition. However, separate models were estimated for the four diffusion metrics.

**Figure 1:**
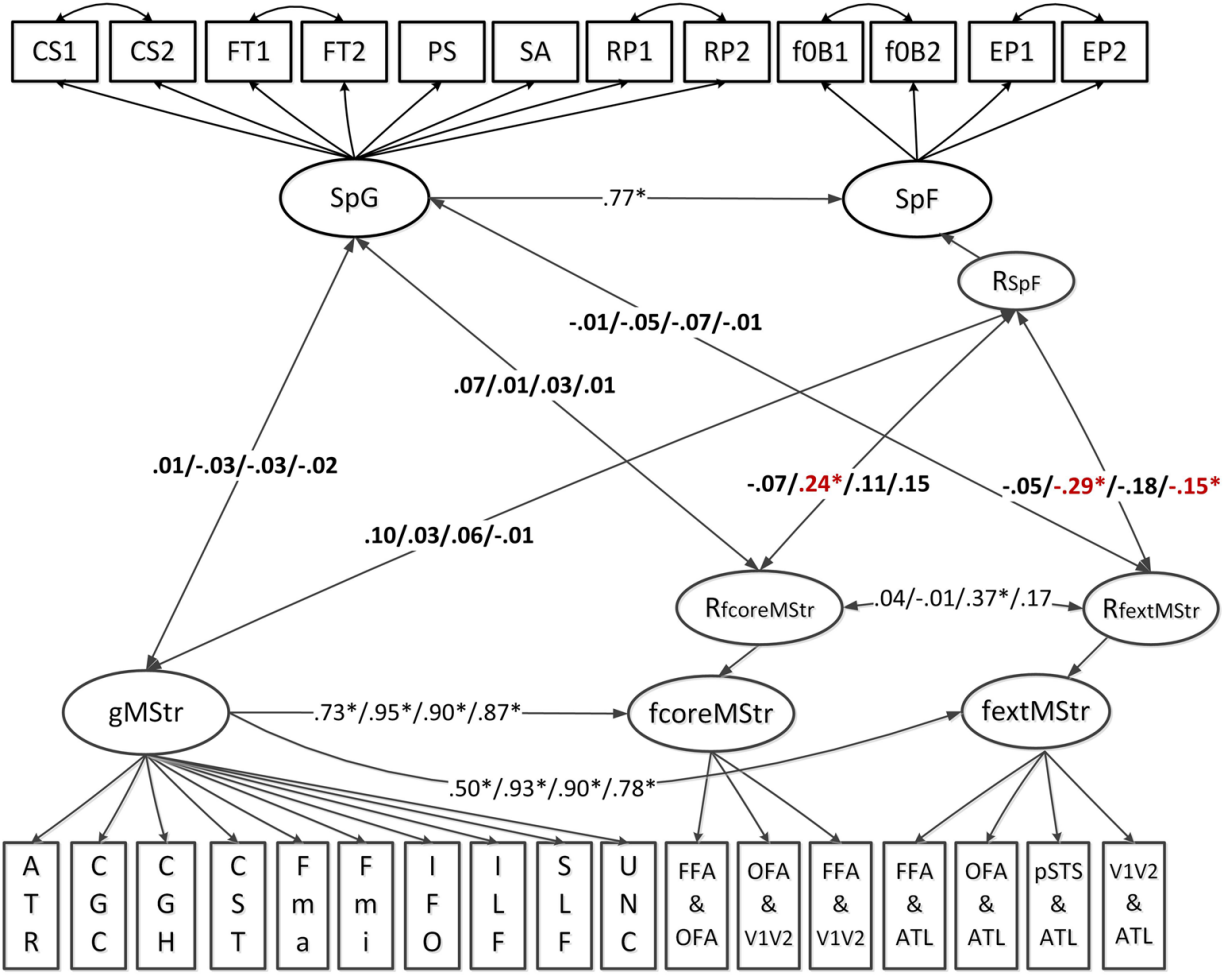
Schematic representation of the structural equation model estimating the linear relationship between individual differences in tract-averaged white matter microstructural properties (FA/MD/AD/RD) and processing speed ability both at the domain general and face-specific levels. SpG—Speed of general cognition; SpF—Speed of face processing; gMStr—Microstructural properties in global brain fibers; fcoreMStr—Microstructural properties of core face network fibers; fextMStr—Microstructural properties of extended face network fibers; RfcoreMStr—Residual variance of the fcoreMStr factor, indicating the unique variance in fcoreMStr not explained by gMStr; RfextMStr—Residual variance of the fextMStr factor, indicating the unique variance in fextMStr not explained by gMStr; RSpF—Residual variance of the SpF factor, indicating the unique variance in SpF not explained by SpG; CS1—Dimensional change card sort task, color domain; CS2—Dimensional change card sort, shape domain; FT1—Flanker inhibitory control and attention task, congruent condition; FT2—Flanker inhibitory control and attention task, incongruent condition; PS—Pattern comparison processing speed; SA—Sustained attention; RP1—Relational processing, first scan as indicator 1; RP2—Relational processing, second scan as indicator 2; f0B1—Facial working memory 0-back task, first scan as indicator 1; F0B2—Facial working memory 0-back task, second scan as indicator 2; EP1—Facial emotion processing, first scan as indicator 1; EP2—Facial emotion processing, second scan as indicator 2. Information on the fiber indicators is provided in Fig. 7. For all the four diffusion metrics, correlated residuals of major fibers included ATR-CGC, ATR-Fmin, CGC-Fmin and UNC-Fmin. Additionally, for the measures of MD, AD and RD, residual covariances of ATR-UNC, SLF-IFO, CGC-UNC, ILF-SLF and ILF-IFO were also estimated.

The white matter and processing speed structural model is displayed in Fig. 1. In this model (see lower part in the model representation), individual differences in white matter microstructural properties of ten global fibers were accounted for by a general latent factor, gMStr. Specific individual differences in microstructural properties in the face network were captured by two further factors, fcoreMstr and fextMstr, respectively. They denote structural connections within the core face-selective brain regions versus the extended face-selective ones. The shared variance of face-specific white matter microstructure scores with those of the global white matter tracts was accounted for by regressing the face-specific factors onto the gMstr factor (see Fig. 1). Residual variances of fcoreMStr and fextMStr are represented by RfcoreMStr and RfextMStr in the figure. They indicate unique variance of white matter microstructure in the core and extended face network that cannot be explained by the global factor. Individual differences in general processing speed (see upper part in the model representation) were captured by the general speed factor SpG. Additionally, a latent factor SpF, measured by multiple face processing tasks, was modeled to account for face specificity in processing speed. Like in the brain model, the shared variance of face-specific speed and general processing speed was controlled for by regressing the SpF factor onto SpG. Thus, the unique face-specific variance is captured by the residual variance denoted RSpF in Fig. 1. As illustrated in the figure, to achieve acceptable fit, several pairs of speed indicator residuals belonging to the same experimental tasks were allowed to correlate. Because these shared variance components are expected by design, residual correlations were deemed an acceptable solution to improve fit. Similarly, residuals of some major white matter indicators were also correlated due to their spatial overlaps revealed by the probabilistic tractography approach^9^. These covariances were not supposed to affect the specific microstructural properties of face network fibers. Standardized factor loadings onto the two latent variables SpG and SpF are provided in Table 1. Factor loadings onto the three white matter factors separately for FA, MD, AD and RD are reported in the supplementary information (see Tables S1 and S2). All factor loadings were statistically significant, but heterogeneous with respect to their magnitude. The model fits were satisfactory, specifically (1) FA-speed model: ${\chi }_{\left(358\right)}^{2}=1084$, $p= .000$, CFI = .95, RMSEA = .04, SRMR = .05; (2) MD-speed model: ${\chi }_{\left(353\right)}^{2}=2488$, $p= .000$, CFI = .91, RMSEA = .077, SRMR = .05; (3) AD-speed model: ${\chi }_{\left(353\right)}^{2}=2571$, $p= .000$, CFI = .91, RMSEA = .078, SRMR = .06; (4) RD-speed model: ${\chi }_{\left(354\right)}^{2}=1676$, $p= .000$, CFI = .93, RMSEA = .06, SRMR = .05. Brain-behavior associations are estimated as correlations at the structural level of the model.

**Table 1:** Standardized factor loadings estimated in the brain-behavior models*.*

Processing speed model			Processing accuracy model		
Task	SpG	SpF	Task	AccG	AccF
CS1	.74		OR	.81	
CS2	.80		VC	.78	
FT1	.68		RAV	.61	
FT2	.67		LS	.44	
PS	.54		SO	.50	
SA	.24		f2B1		.59
RP1	.34		f2B2		.66
RP2	.40		FR1		.25
f0B1		.45	FR2		.25
f0B2		.54			
EP1		.66			
EP2		.58			

All factor loadings are statistically significant (*p* < .05).

*SpG* speed of general cognition, *SpF* speed of face processing, *AccG* accuracy of general cognition, *AccF* accuracy of face processing, *CS1* dimensional change card sort task, color domain, *CS2* dimensional change card sort task, shape domain, *FT1* flanker inhibitory control and attention task, congruent condition, *FT2* flanker inhibitory control and attention task, incongruent condition, *PS* pattern comparison processing speed, *SA* sustained attention, *RP1* relational processing, first scan as indicator 1, *RP2* relational processing, second scan as indicator 2, *f0B1* facial working memory 0-back, first scan as indicator 1, *f0B2* facial working memory 0-back, second scan as indicator 2, *EP1* facial emotion processing, first scan as indicator 1, *EP2* facial emotion processing, second scan as indicator 2, *OR* oral reading recognition test, *VC* vocabulary comprehension, *RAV* Raven progressive matrices, *LS* list sorting working memory, *SO* spatial orientation, *f2B1* facial working memory 2-back task, first scan as indicator 1, *f2B2* facial working memory 2-back task, second scan as indicator 2, *FR1* face recognition, first scan as indicator 1, *FR2* face recognition, second scan as indicator 2.

As illustrated in Fig. 1, SpG and RSpF were correlated with gMStr, RfcoreMStr and RfextMStr to assess the linear relationship between tract-averaged diffusion metric factors and processing speed. Results showed that RfcoreMStr for the MD measure displayed a positive correlation with RSpF (*r* = .24, *p* = .045), meaning that the mean diffusivity of fibers in the core face network was negatively correlated with face processing speed after the effects of both global MD and general processing speed were controlled for. Besides, the RfextMSr for MD and RD were negatively associated with RSpF (*r* = − .29, *p* = .016; *r* = − .15, *p* = .048) when holding two general factors gMStr and SpG constant. Therefore, individual differences in MD and RD of white matter connections in the face network are linearly associated with face processing speed, partialling out the influence of global microstructural properties and general processing speed. Notably, these significant relationships did not emerge without using the semi-partial correlation.

### On the linear relationship between white matter microstructural factors and performance accuracy

**Figure 2:**
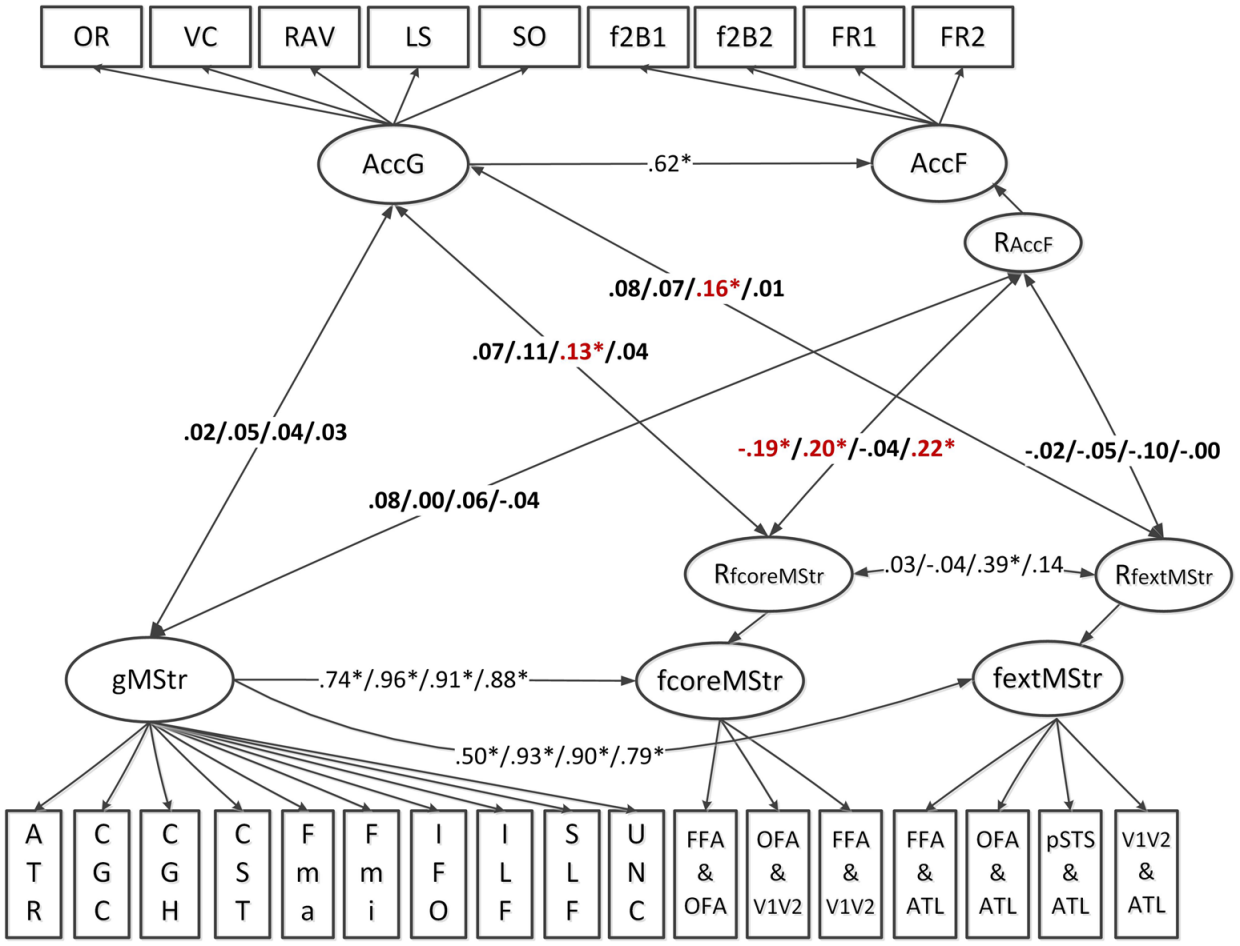
Schematic representation of the structural equation model estimating the linear relationship between individual differences in tract-averaged white matter microstructural properties (FA/MD/AD/RD) and response accuracy at both domain general and face-specific levels. AccG—Accuracy of general cognition; AccF—Accuracy of face processing; gMStr—Microstructural properties in global brain fibers; fcoreMStr—Microstructural properties of core face network fibers; fextMStr—Microstructural properties of extended face network fibers; RfcoreMStr—Residual variance of the fcoreMStr factor, indicating the unique variance in fcoreMStr not explained by gMStr; RfextMStr—Residual variance of the fextMStr factor, indicating the unique variance in fextMStr not explained by gMStr; RAccF—Residual variance of the AccF factor, indicating the unique variance in AccF not explained by AccG; OR—Oral reading recognition test; VC—Vocabulary comprehension; RAV—Raven progressive matrices; LS—List sorting working memory; SO—Spatial orientation; f2B1—Facial working memory 2-back task, first scan as indicator 1; f2B2—Facial working memory 2-back task, second scan as indicator 2; FR1—Face recognition, first scan as indicator 1; FR2—Face recognition, second scan as indicator 2. Information on the fiber indicators is provided in Fig. 7. For all the four diffusion metrics, correlated residuals of major fibers included ATR-CGC, ATR-Fmin, CGC-Fmin and UNC-Fmin. For the measures of MD, AD and RD, residual covariances of ATR-UNC, SLF-IFO, CGC-UNC, ILF-SLF were additionally estimated. Residuals of ILF-IFO, CGC-SLF, Fmin-CGH were also correlated for the MD-accuracy and AD-accuracy model. Fmin-CST and Fmin-ILF were added for AD specifically to improve the model fit.

As for processing speed, we estimated the diffusion metrics (FA/MD/AD/RD) and performance accuracy structural model to assess the relationship between cognitive performance accuracy factors and individual differences in white matter microstructural properties. As illustrated in Fig. 2, the variability in face processing accuracy was accounted for by the AccF factor. Its shared variance with the general processing accuracy factor AccG was accounted for by regressing AccF onto AccG. The unique variance of face processing accuracy is thus denoted as RAccF. Standardized factor loadings onto the two factors AccG and AccF were all significant—they are displayed in Table 1. Factor loadings within the white matter microstructure measurement models were generally equivalent to those estimated in the brain-speed model and in our previous work^9^. They are shown in Tables S1 and S2. The model fits were satisfactory, specifically (1) FA-accuracy model: ${\chi }_{\left(285\right)}^{2}=900$, $p= .000$, CFI = .95, RMSEA = .045, SRMR = .040; (2) MD-accuracy model: ${\chi }_{\left(277\right)}^{2}=2004$, $p= .000$, CFI = .91, RMSEA = .077, SRMR = .049; (3) AD-accuracy model: ${\chi }_{\left(276\right)}^{2}=2074$, $p= .000$, CFI = .91, RMSEA = .079, SRMR = .050; (4) RD-accuracy model: ${\chi }_{\left(281\right)}^{2}=1536$, $p= .000$, CFI = .91, RMSEA = .055, SRMR = .043. The RAccF factor was negatively correlated with RfcoreMStr for FA (*r* = − .19, *p* = .026), and positively correlated with RfcoreMStr for MD (*r* = .20, *p* = .047) and RD (*r* = .22, *p* = .03). Besides, the general accuracy factor, AccG was positively associated with specific AD measures in both core and extended face network, namely RfcoreMStr (*r* = .13, *p* = .018) and RfextMStr (*r* = .16, *p* = .017) in the model. Therefore, we concluded that among young healthy adults, white matter microstructural properties in the face network expressed by FA, MD and RD are correlated with face processing accuracy, accounting for the influence of global fiber structure and general response accuracy.

### On non-linear associations between white matter microstructural factors and cognitive performance

With LSEM, we next explored potential non-linearity of the associations between white matter microstructure (measured by FA, MD, AD and RD) and cognitive ability factors. Estimated parameter gradients of the three white matter factor means across the continuous general and face processing ability scores (factor scores) are computed, with FA means displayed as a representative in the supplementary information (see Figs. S1 to S6). Same as in the linear models, all face network white matter scores were tested for the residual variance (i.e., RfcoreMStr, RfextMStr), in which individual differences in global microstructural properties of the brain are accounted for. Thus, the shared variance between global fibers’ and face fibers’ microstructural properties was partialled out. Behavioral scores of speed and accuracy latent factors were computed in separate measurement models.

Generally, there were barely consistent changes of the mean diffusion metric (FA, MD, AD and RD) factors across processing speed and accuracy levels in both general and face-specific cognitive domains. The pointwise *p*-values provided by the permutation test in LSEM indicate statistical significance (*p* < .05) at different ability ranges in some cases. Apart from the highly heterogeneous trends in the non-linear association functions provided by LSEM, the magnitude of the mean microstructural differences across ability levels was limited. This is in line with a high ratio of null findings of the linear white matter-cognition relationship (see Figs. 1 and 2). Even if we avoid over-interpreting these non-linear associations, for completeness we report the FA results as a representative in the supplement (see Figs. S1 to S6).

### Factorial differentiation of face-network-related versus global white matter microstructure depending on face processing speed and general speed ability

**Figure 3:**
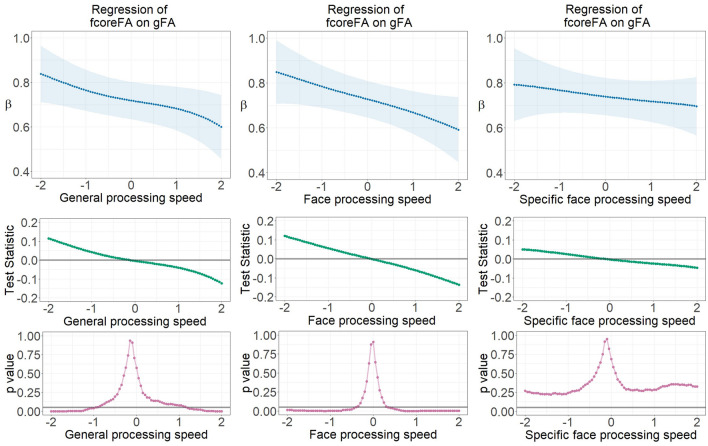
Parameter gradients indicating regressions of fcoreFA on gFA across continuously assessed processing speed ability scores using LSEM, namely (1) general processing speed ability, (2) face processing speed ability, and (3) specific face processing speed ability with general speed controlled for. The upper row displays variations of locally estimated FA factor regressions across processing speed ability scores—FA factor regression parameter functions. The middle row provides the course of the test statistic across processing speed ability scores as estimated with the permutation test. The bottom row displays the pointwise *p*-value curve, with *p* = .05 displayed as a threshold (see the gray horizontal line). β—Standardized regression weight; gFA—Fractional anisotropy in global brain fibers; fcoreFA—Fractional anisotropy of core face network fibers.

**Figure 4:**
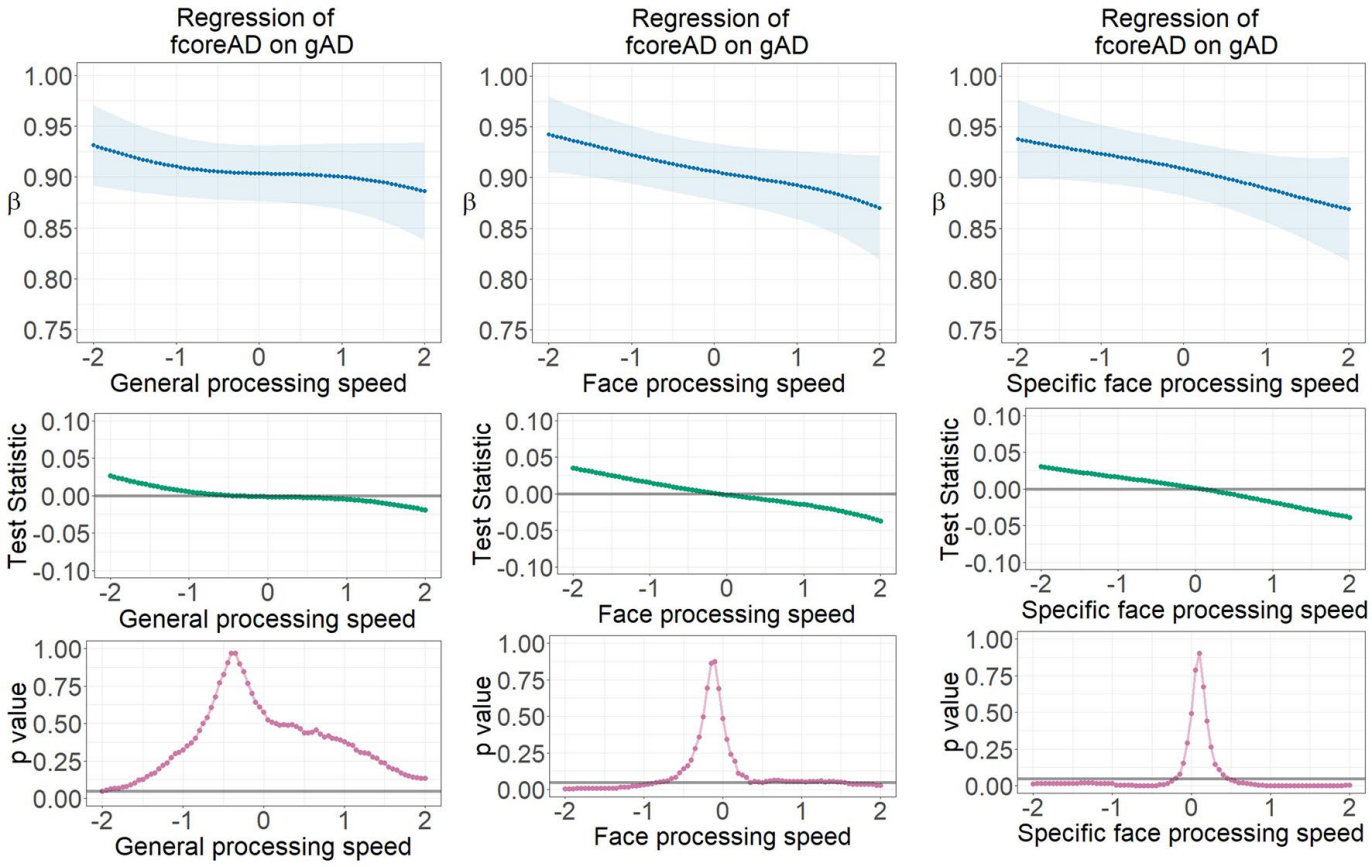
Parameter gradients indicating regressions of fcoreAD on gAD across continuously assessed processing speed ability scores using LSEM, namely (1) general processing speed ability, (2) face processing speed ability, and (3) specific face processing speed ability with general speed controlled for. The upper row displays variations of locally estimated AD factor regressions across processing speed ability scores—AD factor regression parameter functions. The middle row provides the course of the test statistic across processing speed ability scores as estimated with the permutation test. The bottom row displays the pointwise *p*-value curve, with *p* = .05 displayed as a threshold (see the gray horizontal line). β—Standardized regression weight; gAD—Axial diffusivity in global brain fibers; fcoreAD—Axial diffusivity of core face network fibers.

White matter microstructure in the face network and the global region became more differentiated with increasing face processing and general speed ability. This was reflected by decreasing latent regression coefficients between fcoreMStr and gMStr in the FA and AD measures, as well as between fextMStr and gMStr across processing speed ability in the FA measure. Details of the non-linear moderation results are shown in Figs. 3 and 4. LSEM estimated parameter gradients indicating the relationships between every pair of latent white matter factors across continuously assessed face and general processing speed ability scores (factor scores). Specific face processing speed ability (with the influence of general speed ability being partialled out) was also used as a third moderator for the purpose of comparisons.

Specially, each model parameter estimate at a focal point was determined among observations selected by the Gaussian weighting window^30^ moving stepwise along the axis of standardized processing speed scores. All parameter gradients indicate a decrease of the FA or AD factors’ relationships with increasing ability. Thus, in case of individuals with better processing speed ability, the relationship between white matter factors is lower, indicating stronger differentiation of individual differences in global versus face-specific microstructural properties of white matter fiber clusters.

Pointwise *p*-value plots derived from the permutation test generally reveal statistically substantial differentiation at the extremes of low versus high ability range as compared with the average of the entire parameter gradient. The relationship between the global and core face network fibers (fcoreMStr regressed onto gMStr) is largest in the low ability range for both the FA and AD measures. In the FA case, this is not only specific for face processing ability, but it also occurs for general processing speed. However, the statistical significance disappeared after controlling for the influence of the general speed factor. In the AD case, factorial differentiation existed only across face processing speed, no matter if the general speed effect was partialled out.

**Figure 5:**
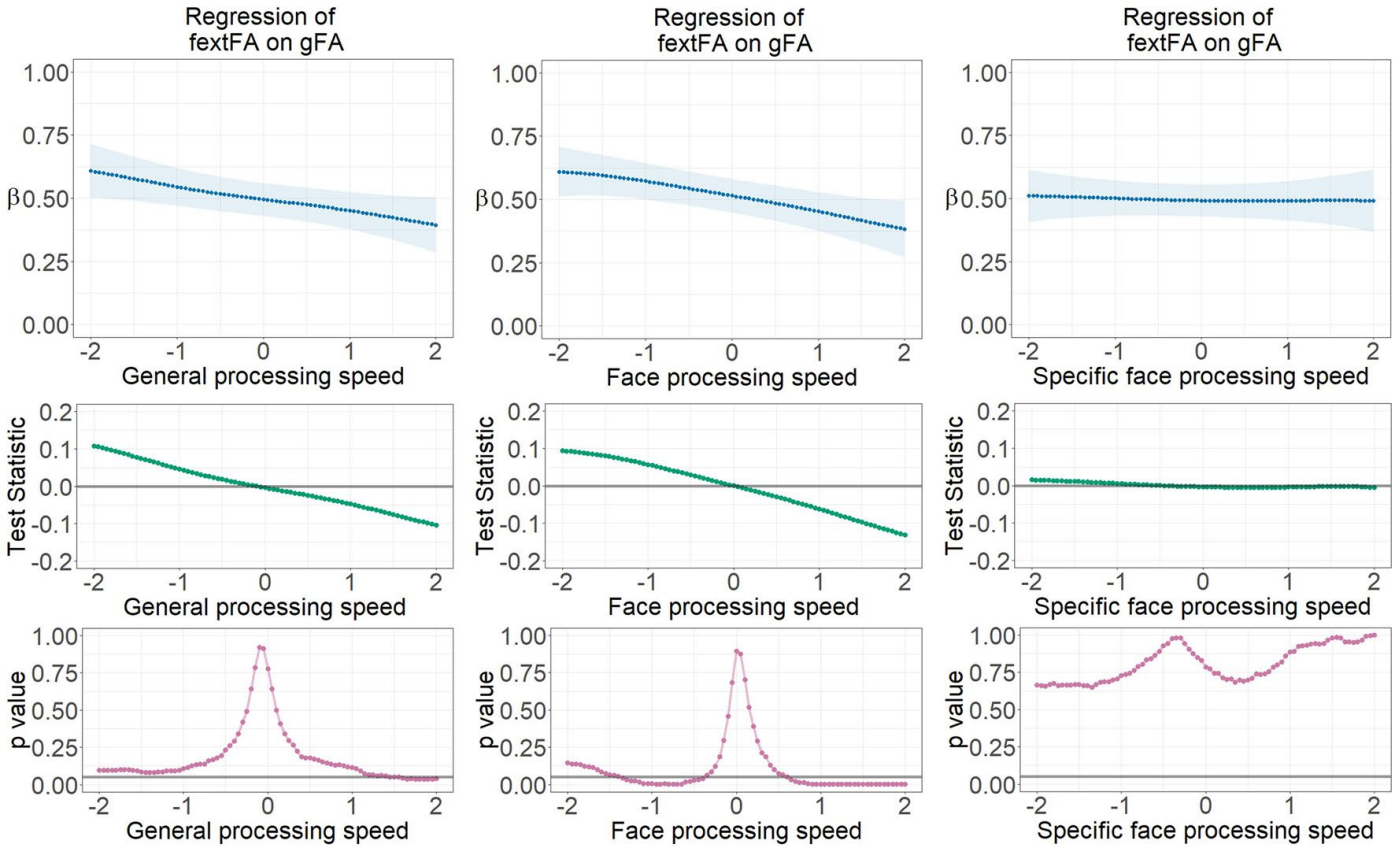
Parameter gradients indicating regressions of fextFA on gFA across continuously assessed processing speed ability scores using LSEM, namely (1) general processing speed ability, (2) face processing speed ability, and (3) specific face processing speed ability with general speed controlled for. The upper row displays variations of locally estimated FA factor regressions across processing speed ability scores—FA factor regression parameter functions. The middle row provides the course of the test statistic across processing speed ability scores as estimated with the permutation test. The bottom row displays the pointwise *p*-value curve, with *p* = .05 displayed as a threshold (see the gray horizontal line). β—Standardized regression weight; gFA—Fractional anisotropy in global brain fibers; fextFA—Fractional anisotropy of extended face network fibers.

The regression weight of the extended face network fibers (fextMStr) on global fibers (gMStr) is significantly lowest at the high ability level for FA only, as shown in Fig. 5. This is again not only true for face processing speed, but it occurs for general processing speed as well. Finally, the parameter functions of the correlation between RfcoreMStr and RfextMstr reveal smallest and non-significant associations, for face processing speed as well as general processing speed.

### Factorial differentiation of face-network-related versus global white matter microstructure depending on face processing accuracy and general cognitive abilities

**Figure 6:**
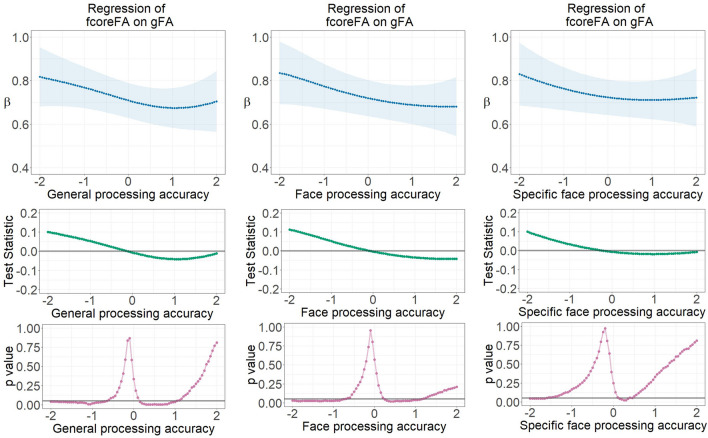
Parameter gradients indicating regressions of fcoreFA on gFA across continuously assessed processing accuracy ability scores using LSEM, namely (1) general processing accuracy ability, (2) face processing accuracy ability, and (3) specific face processing accuracy ability with general response accuracy controlled for. The upper row displays variations of locally estimated FA factor regression coefficients across processing accuracy scores—FA factor relationship parameter functions. The middle row provides the course of the test statistic across response accuracy scores as estimated with the permutation test. The bottom row displays the pointwise *p*-value curve, with *p* = .05 displayed as a threshold (see the gray horizontal line). β—Standardized latent regression weight; gFA—Fractional anisotropy in global brain fibers; fcoreFA—Fractional anisotropy of core face network fibers.

Among all the four white matter microstructural properties, factorial differentiation between face-related and global fibers moderated by response accuracy only showed statistical significance in the FA relationship model. LSEM estimated parameter gradients indicating the regression coefficient between the global and core face network fibers (fcoreFA regressed onto gFA) across continuously assessed face processing accuracy and general cognitive ability scores (factor scores) are displayed in Fig. 6. Generally, the relationship between fcoreFA and gFA decreased along increasing face processing accuracy levels, but such a decrease was also evident for the general cognitive ability.

This means that for individuals with higher cognitive abilities, global and face-specific white matter fiber clusters are more strongly differentiated. Pointwise *p*-value plots reveal statistically substantial differentiation at the low ability range, thus one standard deviation below the sample average regarding face processing accuracy. The relationship between global and face-specific core face network fibers (fcoreFA regressed onto gFA) is largest at the low ability rage. The parameter gradients displaying the correlation between RfcoreFA and RfextFA also show highest values at the lowest ability but lowest values at around average ability, for both face and general processing accuracy. Details are displayed in Fig. S7. For all the other white matter factors, the parameter gradients of factorial relationships do not indicate substantial change across levels of ability. Thus, differentiation between global fibers and face network fibers moderated by response accuracy is only supported by the FA data.

## Discussion

To summarize, we reported a holistic investigation of linear and non-linear relationships of white matter structures in global brain fibers and fibers traced within the face processing brain network with general cognition and face processing related abilities. The results indicate that in young adulthood (1) the tract-averaged MD and RD values of functionally-defined core and extended face fibers are linearly associated with specific face processing speed ability controlled for the effect of general speed ability. (2) the tract-averaged FA, MD and RD values of functionally-defined core face fibers are linearly associated with specific face processing accuracy controlled for the effect of general response accuracy. (3) a factorial differentiation of white matter fiber clusters in the face-selective core brain system and global brain tracts occurs with increasing ability levels.

For the first time in the literature, this study addressed the relationship between white matter structures in the functionally localized face processing brain network and face processing ability at the level of latent variables. This advanced statistical modeling approach enabled us to perform the analysis by taking measurement error into account and avoid confounds due to unreliability of the measurements. In the measurement models of both fiber properties and behavioral performance, domain general and face-specific latent variables were estimated. The estimated latent variables captured a considerable amount of common variance across the observed variables. Importantly, for studying individual differences in face processing brain network specific fiber properties and behavioral performance, we partialled out the shared variance of the microstructural properties of face-specific fibers with the global fibers’ properties. Therefore, the accumulated results can be interpreted as specific for faces. This control for the positive manifold turned out to be beneficial and theoretically relevant in previous studies as well^4^.

There are two influential individual differences studies in the literature which reported substantial associations between FA in global brain fibers and general intelligence^31,32^. These studies indeed inspired the present work. However, we expected divergent results for the young adult HCP sample at least for the general cognition domain. This is because we suspected the reported relationships in those studies to be due to the age heterogeneity of their samples. In young adults between 22 and 35 years of age, it can be concluded that white matter fiber properties are not directly associated with cognitive performance. Due to the large sample size involved in our analyses, this conclusion can be made with large confidence.

The divergent findings compared with those previous reports^31–33^ can be explained by the broader ability range, including variation between individuals and across age, for which the conclusions of an association can be drawn. Previous brain-behavior studies which reported significant linear associations between microstructural properties of global white matter fibers and cognition, either involved wider life-span samples or patient, thus individuals from the far lower ability range and thus larger individual differences. For example, lesions in brain fibers were found to correlate with impairments in executive function^34^, episodic memory^35^ and psychomotor speed^36^.

In healthy individuals, it is well-established that children^37^ and elderly^38^ undergo intensive changes and transformations also with respect to microstructural properties of brain fibers as well as in cognition. However, in young adulthood where abilities vary the least, a linear association between fiber properties and domain general cognitive abilities does not seem to exist. Hitherto proposed theoretical accounts are in line with this interpretation. For example, Johnson and colleagues^39^ theorized and demonstrated that age was a relevant mediator between properties of white matter and motor speed. Another study on adolescents and young adults showed that the FA relationship with delayed discounting behavior was stronger in adolescents (9–16.5 years old) as compared with young adults (16.5–23 years old)^40^. Furthermore, it has been shown that the FA of white matter structures consistently increases across childhood development, remains rather stable in young adulthood and decreases in elderly, reflecting brain structure development and degeneration^41,42^. Therefore, it is plausible to assume that individual differences in microstructural white matter properties among young healthy adults are not strong enough to be predictive of cognitive ability.

After partialling out the effects of global white matter properties and general cognitive ability, significant relationships were shown between specific face network fibers and specific face processing abilities. This reflects the importance to consider the influence from general factors in the estimation of brain-behavior relationship, which was proposed and proved by a series of previous studies in the face processing domain^1–5,9^. As shown in Figs. 1 and 2, the FA, MD and RD measures displayed a high sensitivity of brain microstructural change and its covariation with face-specific cognitive performances, as compared with AD. This is consistent with several relevant findings. For example, in a developmental study, Sherf and colleagues (2014) found age-related differences in the FA, MD and RD but not AD mean values of the global fiber ILF, which was specifically related with a size increase of the face-selective brain functional region^43^. Besides, these three diffusion metrics also showed significant correlations with the face processing performance of individuals with autism spectrum disorder and with domain-specific traumatic brain injury^44,45^. Notably, as suggested by Gomez et al. and Jones et al.^15,19^, the interpretation of diffusion MRI metrics should be very careful since an increase or decrease of the value does not simply indicate a positive or negative change of the white matter quality. Furthermore, the factorial relations in the linear models must be interpreted with caution when either covariate has only little variance, as is for example the case with the specific connectivity factors of MD and AD that load on their respective general connectivity factor with β ≥ .90. However, a key message is straightforward—the anatomical connectivity in the individualized local face network plays an important and specific role in supporting individual differences of face processing ability.

Locally estimated fiber microstructure in the core and extended face processing brain network, after partialling out the shared variance with the global fibers’ properties did not show a similar consistent change across ability. Global brain fiber properties instead displayed a comparably steadier change across the entire ability range of face processing speed and accuracy. Generally, only very few previous studies investigated the relationship between functionally defined brain fibers in the face processing network and face processing ability, especially from the non-linear perspective. Gomez and colleagues^19^ used data from only 18 adults aging between 18 and 40 years. They defined only one face-selective fiber intersecting a spherical functional ROI in the FFA-2 area and an anterior anatomical plane. Although the properties of that specific face-selective fiber were shown to significantly correlate with face processing performance, approaches in Gomez et al. were largely different from the designs and methods we used in the current investigation.

In a more recent study, Wang and colleagues explored the brain anatomy-function relationship in face processing based on the HCP data^46^. They also tested whether the regional and global connectome features can predict the facial emotion recognition ability. Simple Pearson correlation analysis didn’t show any significant results between facial emotion recognition and all brain neural features, including the FA, MD, AD and RD values of 92 white matter tracts. A machine learning approach followed by revealed a small subgroup of predictive white matter FA values, including two global fibers and four regional fibers in the face processing brain network. This not only indicated the diverse roles of fibers in explaining individual differences in face processing, but also reflect the importance of finding a good way to reveal features of brain or behavior in a specific cognitive domain. Therefore, it is of great value in the current study to control for the effect of global white matter properties and general cognitive ability in the face processing related analysis.

Differentiation of individual differences with respect to the structural properties of the face processing brain network has not been studied before. An age-homogenous sample of young healthy adults allows a best estimate of differentiation because variation due to developmental, aging and atypical conditions are ruled out. We discovered that the relationships between global brain and face-specific white matter fiber properties are modulated by ability level. More specifically, our study shows that individual differences in the white matter properties of global and face-specific fiber clusters are more differentiated at higher levels of face processing and general cognitive ability. The moderation effect is not face processing ability specific, but it also holds for general cognition speed and accuracy. However, it is somewhat stronger in case of face cognition as compared with general cognitive ability.

To the best of our knowledge, previous studies have never provided direct evidence about ability dependent differentiation of brain white matter structure in the face processing domain. However, since individuals’ cognitive abilities gradually deteriorate and de-differentiate across adult lifespan^29^, we can interpret our findings in the light of the studies about age differentiation and de-differentiation. For example, based on 3513 participants from the UK Biobank, Cox and colleagues found that the microstructure of 27 major white matter tracts showed increasing correlations (i.e. de-differentiation) from 45 to 75 years, measured by tract averaged FA, MD and other diffusion metrics^47^. This de-differentiation of white matter structure across age groups is a strong support to our ability-related result considering the well-known relationship between aging and cognitive deterioration^29^. In another study, the relationships between a global white matter latent factor and tract-averaged FA of major tracts displayed mixed patterns in the age range between 18 and 88 years^42^. Specifically, these factor loadings showed various fluctuations before 60 years of age across different fibers, which may partially explain the nonmonotone non-linear brain-behavior results in the current study. Importantly, after 60 years of age, when cognitive abilities usually exhibit a steeper decrease^29^, all the factor loadings started to jointly increase, indicating a dedifferentiation between microstructural features of single fibers and global white matter property. This may to some extent account for the dedifferentiation of white matter clusters among the cohort of individuals with lower cognitive levels, as shown in our current finding. Future studies will need to uncover the cellular and metabolic underpinnings behind this interesting brain structural differentiation-dedifferentiation phenomenon discovered at the level of individual differences.

The present findings complement previous reports on functional differentiation in the face processing brain network^24,48^ with elaborated neuroanatomical support for differentiation. The described findings are in line with the reported structural–functional correspondence in the face processing brain as well^9,11,19^. In previous research on face processing functions, a so called brain adaption theory has been proposed^25^. According to this theory, age-related decline in the brain’s functional specificity regarding face processing is compensated by global brain areas, such as the prefrontal cortex. This compensation guarantees successful neural processing of faces. fMRI data from about three hundred individuals between 20 and 89 years of age revealed face-selective brain functional regions such as the FFA to become less responsive to face stimuli with increasing adult age^24^. Furthermore, face-selective areas exhibited stronger activation when processing other types of non-face stimuli. Developmental studies addressed functional differentiation in the face processing brain regions by comparing young adults with children^26,49,50^. Using topological methods, functional connectivity in the face network activated by a face processing task was found to be elevated in young adults as compared with children. Furthermore, it has been repeatedly shown that neural differentiation-dedifferentiation across development and aging is closely associated with age-related changes of cognitive performance^51,52^. Specifically, the strongest functional differentiation in the face network can be observed in young adults which is representing the peak of cognitive performance across the life span. In line with the above fMRI evidence, our findings demonstrate face specificity at the neuroanatomical level which is pronounced in case of individuals with higher ability levels. Differentiation occurs between face-selective fibers and global brain white matter fibers, and it is most robust across different ability domains with respect to the core face network and global fibers with increasing ability. Note that the anatomical differentiation in the face network is not entirely face processing ability specific, but it also holds for general cognitive performance according to a similar pattern. This might be a substantial finding or a methodological bias to some extent. We speculate so, because the assessment paradigm of face processing ability applied in HCP has a strong working memory load (see Van Essen et al.^53,54^ and the task description above). This leads to a very high correlation between the estimates of face processing and general cognitive ability. Should the face processing tasks capture pure face perception ability, the face-specificity of the moderation effect might turn out to be stronger.

Among the few studies dedicated to investigate brain structural underpinnings of functional differentiation-dedifferentiation in face processing, Rieck and colleagues^51^ reported a significant relationship between the white matter microstructural properties of the inferior longitudinal fasciculus (ILF) and functional dedifferentiation in face-specific response in an age heterogeneous sample. Specifically, degradation of the ILF in normal aging was associated with less functional sensitivity and selectivity to face stimuli in the FFA. Our study focused on another age range and multiple fibers and addressed differentiation of individual differences in anatomical properties of the brain, thus being complementary to Rieck et al.^51^.

The described findings of this study are in line with Spearman’s Law, according to which at lower ability levels cognitive functioning is more strongly supported by general resources, whereas at higher ability levels cognitive functions are more constrained by domain specific resources indicating stronger independency^29,55^. The current findings validate and further extend Spearman’s law from the perspective of brain structural organization by revealing that ability-related differentiation does not only occur at the behavioral level among diverse cognitive domains, but also at the brain’s anatomical level among global and functionally specific white matter structures.

The HCP dataset is highly valuable and it allows large scale investigations of brain-behavior associations for understanding human cognition. The large sample also guarantees robust estimates of associations and prevents false discoveries due to low statistical power. With the provided multimodal brain data, researchers are provided with a unique opportunity to explore the neural correlates of cognition. However, though comprehensive for such a data resource, some compromises had to be made at the level of the collected behavioral data in the HCP. Thus, domain specific abilities such as face processing were assessed with a very few tasks only (partly in the scanner), and the number of the applied trials within a task was rather low. Estimated face processing factors in the present study are thus rather task specific and future studies are needed for replication with more comprehensive domain specific task batteries to prove generalizability of the present findings based on multivariate assessments. Besides, the HCP preprocessing pipelines have improvement options in the future as compared with emerging new algorithms in recent years. Independently of these limitations, the present results are seminal, with the potential to guide attention toward the investigation of differentiation of individual differences in anatomical structures across ability levels for a better understanding of domain specific versus domain general human cognitive abilities and their underlying white matter microstructural properties.

Taken together, in large sample of young healthy adults, we investigated whether microstructural properties of fibers within the face processing brain network reveals neuroanatomical specialization with increasing face processing and general cognitive ability. We found that the white matter microstructural properties (FA, MD and RD) of functionally-defined fibers in the face processing brain network are linearly associated with domain specific cognitive abilities after controlling for the general brain and cognitive effect in young adulthood. Besides, with higher ability, individual differences in properties of white matter fiber clusters of the core face network are more strongly differentiated from global brain fibers, indicated by a lower relationship. These findings emphasize a new perspective on the study of neural structure and function supporting domain specific and domain general abilities.

## Methods

### Participants

All participants were young healthy adults from the WU-Minn Human Connectome Project (HCP; www.humanconnectome.org/study/hcp-young-adult), a multimodal database available for public scientific use^53,54^. In the current study, we involved all participants who completed the diffusion MRI (dMRI) measurements, along with a working memory task with faces during an fMRI recording and a psychometric task battery outlined below. The sample with valid data to address associations of dMRI measures and processing speed abilities contained of *N* = 1031 participants (553 females, 22–35 years of age). For modelling brain-behavior associations in the domain of performance accuracy, a total sample of *N* = 1025 participants (550 females, 22–35 years) was available in the HCP dataset. Informed consent was obtained from each participant. The Institutional Review Board (IRB) of Washington University approved the HCP study^53,54^. All experiments were performed in accordance with relevant guidelines and regulations.

### MRI data processing

The HCP database provided high quality brain images in different modalities (structural, functional and diffusion MRI), collected by a customized Siemens 3T at Washington University in St. Louis^53,54^. The T1-weighted (T1w) structural data were scanned with a 0.7 mm isotropic resolution (TR = 2400 ms, TE = 2.14 ms, TI = 1000 ms, Flip Angle = 8 deg, FOV = 224). The diffusion images were obtained at a 1.25 mm spatial resolution with three shells (b = 1000, 2000, 3000 s/mm^2^) and 270 directions equally allocated in each shell. The functional brain data were sampled using a 2 mm resolution (TR = 720 ms, multiband factor = 8, 1200 points per run).

All the multimodal raw data were preprocessed by a specifically designed minimal preprocessing pipeline, including basic sequential steps of artifact and distortion removal, cortical surface generation, cross-modal registration, and so on^56^. Besides, task-evoke fMRI data were further analyzed to generate between-condition contrasts^57^. Prerequisite fiber orientation estimation parameter files named “BEDPOSTX” were also generated. All the above preprocessed and partially analyzed MRI data are provided by HCP experts^56,57^.

To investigate the relationship between white matter microstructural property both within the face selective brain network and for global brain fibers and behavioral performance, we traced seven fibers linking functionally defined face areas and ten major white matter bundles. The detailed procedure has been recently outlined in our previous work^9^. Here we provide a brief description. In the face processing brain network^6,7^, six regions of interest (ROIs), namely the FFA, OFA, pSTS, ATL, IPS and the early visual retinotopic regions (V1-V2) were localized for each individual using the Connectome Workbench software (https://www.humanconnectome.org/software/connectome-workbench)^51^. Within the anatomically located brain regions^9^, each individualized surface ROI was drawn around the peak vertex with a geodesic radius of 10 mm. This cortical vertex was activated strongest by the face stimuli as it was indicated by the *face versus average* contrast map measured during the HCP working memory scanner task with faces and other objects as stimuli.

Probabilistic tractography was applied for fiber reconstruction using the FMRIB Software Library (FSL version 5.0.9; fsl.fmrib.ox.ac.hk)^58,59^. White matter fibers were traced between each pair of face selective ROIs in both hemispheres based on FMRIB’s Diffusion Toolbox (FDT) in the FSL software^58,59^. Bidirectional fiber tracing was performed using one ROI as the seed region and the other as the target, then vice versa. Default settings in the FDT were applied with stop masks given by brain pial surface and voxels whose FA values lower than 0.1. Similar approaches were also applied in face processing studies before^11,16,19^. A group of comparably strong white matter links within the face processing brain network were selected for solid statistical modeling, namely the FFA-OFA, OFA-V1V2, FFA-ATL, V1V2-ATL, OFA-ATL, pSTS-ATL and FFA-V1V2 connections^9^.

In a standard two-ROI procedure, two anatomically defined reference ROI slices for each global fiber were transformed from the standard MNI space to individual diffusion space^9^. The following ten global fibers were traced by means of probabilistic tractography: the anterior thalamic radiation (ATR), cingulum in the cingulate gyrus part (CGC), the cingulum in the hippocampal part (CGH), the corticospinal tract (CST), the inferior fronto-occipital fasciculus (IFO), the inferior longitudinal fasciculus (ILF), the superior longitudinal fasciculus (SLF), the uncinate fasciculus (UNC), the occipital projection of the corpus callosum (forceps major, Fmajor), and the frontal projection of the corpus callosum (forceps minor, Fminor).

**Figure 7:**
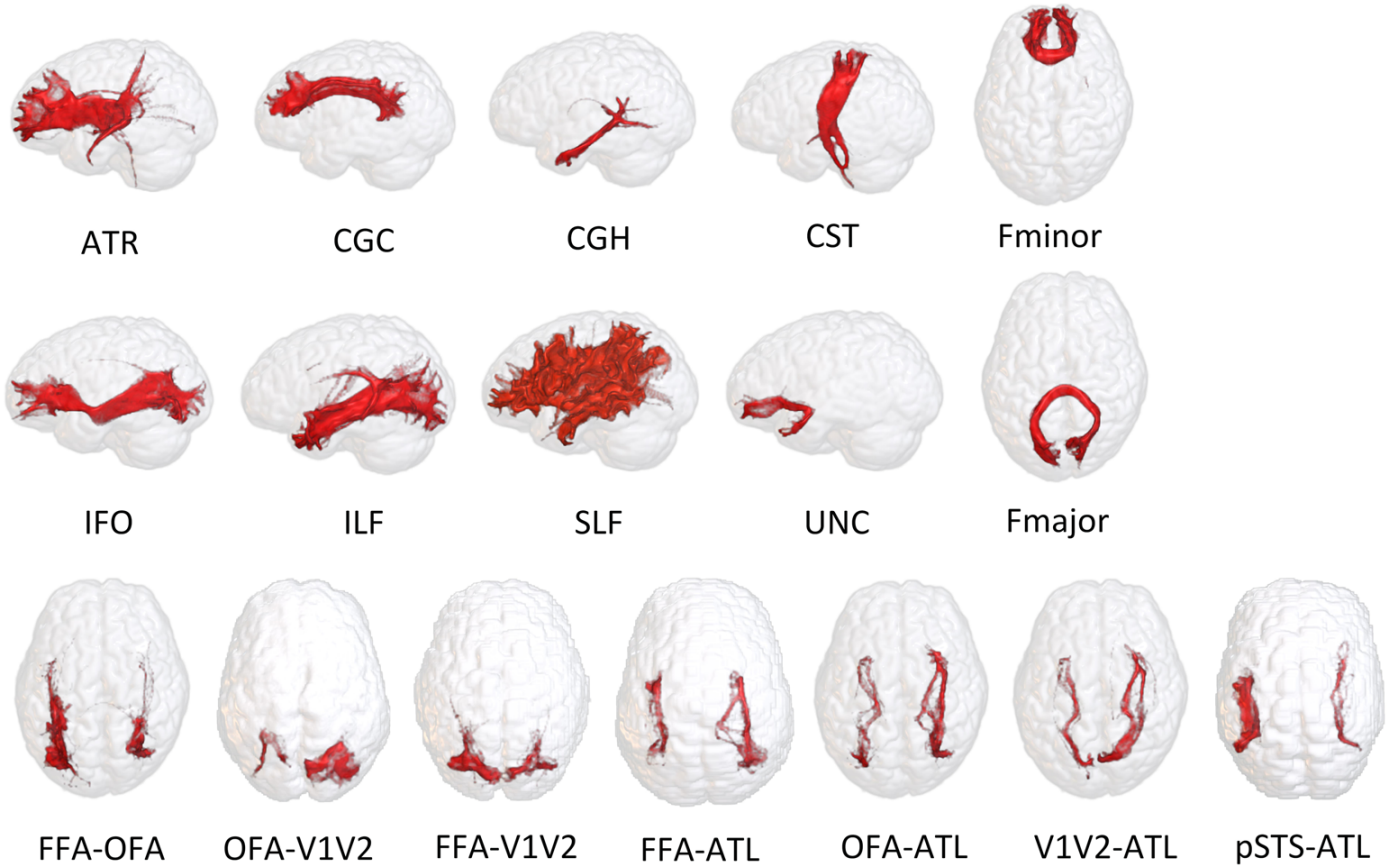
Ten global fibers and seven functionally defined face fibers from representative individual brains traced in the current study. Displayed individuals were selected according to their white matter connection to provide a clear visualization of the traced fibers. ATR—anterior thalamic radiation; CGC—cingulate gyrus part; CGH—cingulum in the hippocampal part; CST—corticospinal tract; IFO—inferior frontooccipital fasciculus; ILF—inferior longitudinal fasciculus; SLF—superior longitudinal fasciculus; UNC—uncinate fasciculus; Fmajor—occipital projection of the corpus callosum (forceps major); Fminor—frontal projection of the corpus callosum (forceps minor); FFA—fusiform face area; OFA—occipital face area; pSTS—posterior superior temporal sulcus; ATL—anterior temporal lobe; V1V2—early visual retinotopic regions.

All these brain fibers originating from representative individuals are visualized in Fig. 7. Four diffusion MRI metrics, namely fractional anisotropy (FA), mean diffusivity (MD), axial diffusivity (AD) and radial diffusivity (RD) were used as measure of alternations in white matter microstructural properties^31,60^. For each global and functionally-defined face fiber, the tract-averaged FA, MD, AD and RD values were computed using the FDT tool of the FSL software. Fibers located in both hemispheres were weighted in their average computation.

### Psychometric tasks

In the HCP study, cognitive abilities were assessed in various content domains. Some tasks of interest for the present study were administered during fMRI, whereas other psychometric tasks^57^ were acquired outside the scanner. Some tasks targeted performance speed, others performance accuracy, providing a contrast between easy and difficult tasks^1,3,4,61,62^. Here, we aimed to model individual differences in microstructural properties of multiple traced fibers in their relationship with performance speed, as well as performance accuracy. Seven tasks were thus selected from the HCP database which allow to indicate different facets of processing speed abilities, namely Dimensional Change Card Sort (CS), Flanker Inhibitory Control and Attention Task (FT), Pattern Comparison Processing Speed (PS), Sustained Attention (SA), Relational Processing (RP), Working Memory facial 0-Back (f0B) and Emotion Processing (EP). Similarly, we selected seven accuracy tasks, capturing performance under rather difficult demands, namely Oral Reading Recognition Test (OR), Vocabulary Comprehension (VC), Raven Progressive Matrices (RAV), List Sorting Working Memory (LS), Spatial Orientation (SO), Working Memory facial 2-Back (f2B), Face Recognition task (FR). Among the selected tasks, two were collected during the scanning session. A brief description of each task is provided in the supplementary information.

### Behavioral data preprocessing

Univariate distributions of reaction times (RTs) and accuracy across participants in each task were visually inspected. Out of the relational processing task, we retained the control matching condition and removed the relational processing condition since its difficulty was too high for a speed task according to the observed performance distributions. All other distributions revealed appropriate score dispersion for speed or accuracy indicators. Note that accuracy in speed tasks should be at ceiling.

A strict preprocessing was carried out for all speed tasks. First, trial-level RTs below 200 ms were excluded, because these reactions were too fast to reflect valid responses. Second, the average RT across trials was computed for each individual by taking only correct responses into account. Third, for participants with an accuracy below 60% in easy speed tasks, the RT average score was set to missing. Further, outliers in each between person RT distribution (> 3.5 *SD*) were excluded iteratively. The percentage of data cells set to missing based on these criteria was 3.2%. Participants with missing values in five or more tasks were excluded completely. Specifically, only one participant was eliminated due to much too fast RTs (< 200 ms) and no variation across trials. Finally, all RTs (in millisecond) were transformed into inverted latency scores (1000/RT). These scores indicate the number of correct trials per second and are in line with accuracy scores with respect to their dimensionality (the higher the better for both measures).

In accuracy tasks, the proportion of correct responses were computed with non-response set to missing. Three tasks from the NIH toolbox, namely oral reading, vocabulary comprehension and working memory list sort were assessed by test administers^63^. We chose the age-adjusted scores of these three tasks provided by the HCP, indicating performance among individuals of the same age at a national range. All preprocessing steps were conducted in the R Software^64^.

### Statistical analysis

Structural Equation Modeling (SEM)^65^ was applied to investigate linear brain-behavior associations of diffusion metrics (FA, MD, AD and RD) belonging to different brain fibers and cognitive abilities. As reported in our previous work^9^, individual differences in FA of global fibers can be explained by a general factor (gFA, global fractional anisotropy). While the microstructural property (FA) of functionally-defined fibers in the face network can be accounted for by specific factors for the core face network (fcoreFA; corresponded to structural connections between FFA, OFA and V1V2) and the extended face network (fextFA; corresponded to structural connections from ATL to FFA, OFA, pSTS and V1V2). In the current study, we aim to relate a measurement model of performance speed and accuracy with the measurement model of the white matter microstructure established in our previous work (see Figs. 1 and 2). We extended the previous FA measure to four diffusion metrics namely FA, MD, AD and RD, and introduced the abbreviation MStr (for “MicroStructure”) in the model as a collective name for the four metrics. Instead of using a nested bi-factor minus-one model as in Liu et al.^9^, we estimated three white matter microstructure factors based on specific indicators (i.e., gMStr, fcoreMStr and fextMStr, no cross loadings). However, we estimate the shared variance of fcoreMStr and fextMStr with gMStr as in our previous work, by regressing fcoreMStr and fextMStr onto gMStr. This factorial representation facilitates factorial differentiation analysis. The latent factor gMStr captures the shared variance of tract-averaged white matter microstructural properties (i.e. FA, MD, AD and RD) in ten global brain fibers. The latent variable fcoreMStr accounts for the common variance in diffusion metric values of fibers in the core face processing brain network. In the described models, we regress fcoreMStr onto gMStr to investigate whether individual differences in properties of the face network fibers are differentiable from the properties of global fibers. This asks whether global fiber quality explains the variation in face related fibers.

We further aim to investigate non-linear associations between white matter factors and face processing ability. To this purpose, we applied Local Structural Equation Modeling (LSEM)^30^, in which face processing speed and accuracy are treated as continuous moderators of the white matter microstructural measurement model. Factor scores of processing speed and accuracy were computed with the lavaan package in R^64^ and used as continuous moderators in separate LSEMs. In a nutshell, LSEM was proposed to locally estimate a measurement or structural model at discrete values of a continuous moderator by applying a moving weighting window of observations along the moderator. With this procedure, it allows to flexibly explore the parameter change in a measurement model across the continuous moderator.

Here, we investigated two classes of LSEM parameter estimates of the fiber microstructure measurement model (correlated factors of gMStr, fcoreMStr and fextMStr) with respect to their parameter gradient along both general and face processing speed versus accuracy. First, we inspected the latent mean gradient of global and face-specific MStr factors across ability scores. Next, we analyzed the MStr factors’ relationship gradient across processing speed and accuracy aiming to investigate whether the structure of individual differences in global and face-specific MStr factors differentiate with increasing ability. Face processing abilities with and without controlling for the general performance effect were both applied as moderators. Permutation tests (*n* = 500) were carried out in order to evaluate the significance of parameter deviations from the average parameter estimate^30^.

Model fit in SEM and LSEM was evaluated by the χ2-goodness of fit, the comparative fit index (CFI), the root mean square error of approximation (RMSEA) and the standardized root mean-square residual (SRMR)^66^. An acceptable model fit should achieve a CFI value larger than 0.95, as well as both SRMR and RMSEA values smaller than 0.08^67^. All statistical analyses were conducted with the lavaan and sirt package^68^ in the R software^64^. All the analysis scripts have been uploaded to the third-party repository OSF. They can be accessed via the following link: https://osf.io/udqw3/.

## Supplementary Information

Supplementary Information.
