# Supplementary material for: Individual differences in white matter microstructure of the face processing brain network are more differentiated from global fibers with increasing ability
Source: Sci Rep. 2022 Aug 18;12:14075. doi: 10.1038/s41598-022-17850-4 (PMC9388653; doi:10.1038/s41598-022-17850-4)
Supplement: Supplementary file 1 — Supplementary Information. [file 41598_2022_17850_MOESM1_ESM.pdf]

Supplementary Information

Individual differences in white matter microstructure of the face processing brain network are more differentiated from global fibers with increasing ability

Xinyang Liu[1,2,3*], Mattis Geiger[4,5], Changsong Zhou[3], Andrea Hildebrandt[2,6*]

[1] Shanghai Key Laboratory of Brain Functional Genomics (Ministry of Education), School of Psychology and Cognitive Science, East China Normal University, Shanghai, 200062, China

[2] Department of Psychology, Carl von Ossietzky University of Oldenburg, 26129, Germany

[3] Department of Physics, Centre for Nonlinear Studies, Institute of Computational and Theoretical Studies, Hong Kong Baptist University, Hong Kong

[4] Institute of Psychology and Education, University of Ulm, 89069, Germany

[5] Department of Implementation Research, Bernhard Nocht Institute for Tropical Medicine, 20359 Hamburg, Germany

[6] Research Center Neurosensory Science, Carl von Ossietzky University of Oldenburg, 26129, Germany

***** Correspondence should be addressed to:

Xinyang Liu
East China Normal University
School of Psychology and Cognitive Science
Zhongshan Road N. 3663
200062, Shanghai, China
Tel: +86 (0)21 62232963
Email: xyliu@psy.ecnu.edu.cn

Andrea Hildebrandt
Carl von Ossietzky University of Oldenburg
Department of Psychology
Ammerländer Heerstr. 114-118
26129 Oldenburg, Germany
Tel: +49 (0)441 798-4629
Email: andrea.hildebrandt@uni-oldenburg.de

Fiber	White matter microstructure - Speed Model		
	**gMStr** (FA/MD/AD/RD)	**fcoreMStr** (FA/MD/AD/RD)	**fextMStr** (FA/MD/AD/RD)
**ATR**	.80/.76/.74/.79		
**CGC**	.73/.72/.73/.73		
**CGH**	.50/.86/.89/.66		
**CST**	.68/.83/.83/.70		
**Fma**	.62/.79/.81/.64		
**Fmi**	.64/.43/.39/.60		
**IFO**	.93/.84/.84/.90		
**ILF**	.94/.96/.95/.98		
**SLF**	.90/.83/.80/.91		
**UNC**	.69/.68/.66/.74		
**FFA-OFA**		.54/.82/.90/.62	
**OFA-V1V2**		.50/.76/.72/.62	
**FFA-V1V2**		.40/.32/.33/.37	
**FFA-ATL**			.64/.52/.55/.55
**OFA-ATL**			.86/.72/.72/.75
**pSTS-ATL**			.27/.22/.25/.15
**V1V2-ATL**			.49/.64/.63/.68

Table S1. Standardized factor loadings of white matter factors estimated in the brain -speed models. All factor loadings were statistically significant ($p < .05$). ATR – anterior thalamic radiation; CGC – cingulate gyrus part; CGH – cingulum in the hippocampal part; CST – corticospinal tract; IFO – inferior fronto-occipital fasciculus; ILF – inferior longitudinal fasciculus; SLF – superior longitudinal fasciculus; UNC – uncinate fasciculus; Fmajor – occipital projection of the corpus callosum (forceps

major); Fminor – frontal projection of the corpus callosum (forceps minor); FFA – fusiform face area; OFA – occipital face area; pSTS – posterior superior temporal sulcus; ATL – anterior temporal lobe; V1V2 – early visual retinotopic regions.

**Fiber**	**White matter microstructure - Accuracy Model**		
	**gMStr**	**fcoreMStr**	**fextMStr**
	**(FA/MD/AD/RD)**	**(FA/MD/AD/RD)**	**(FA/MD/AD/RD)**
**ATR**	.80/.75/.75/.79		
**CGC**	.73/.73/.76/.73		
**CGH**	.50/.85/.87/.66		
**CST**	.67/.82/.82/.70		
**Fma**	.62/.80/.81/.65		
**Fmi**	.64/.45/.47/.60		
**IFO**	.93/.86/.87/.90		
**ILF**	.94/.97/.96/.99		
**SLF**	.90/.84/.83/.91		
**UNC**	.69/.69/.67/.73		
**FFA-OFA**		.53/.81/.89/.62	
**OFA-V1V2**		.51/.77/.73/.62	
**FFA-V1V2**		.39/.35/.35/.38	
**FFA-ATL**			.61/.49/.52/.53
**OFA-ATL**			.86/.73/.73/.75
**pSTS-ATL**			.26/.22/.24/.16
**V1V2-ATL**			.50/.65/.63/.69

Table S2. Standardized factor loadings of white matter factors estimated in the brain - accuracy models. All factor loadings were statistically significant ($p < .05$). ATR – anterior thalamic radiation; CGC – cingulate gyrus part; CGH – cingulum in the hippocampal part; CST – corticospinal tract; IFO – inferior fronto-occipital fasciculus; ILF – inferior longitudinal fasciculus; SLF – superior longitudinal fasciculus; UNC – uncinate fasciculus; Fmajor – occipital projection of the corpus callosum (forceps major); Fminor – frontal projection of the corpus callosum (forceps minor); FFA – fusiform face area; OFA – occipital face area; pSTS – posterior superior temporal sulcus; ATL – anterior temporal lobe; V1V2 – early visual retinotopic regions.

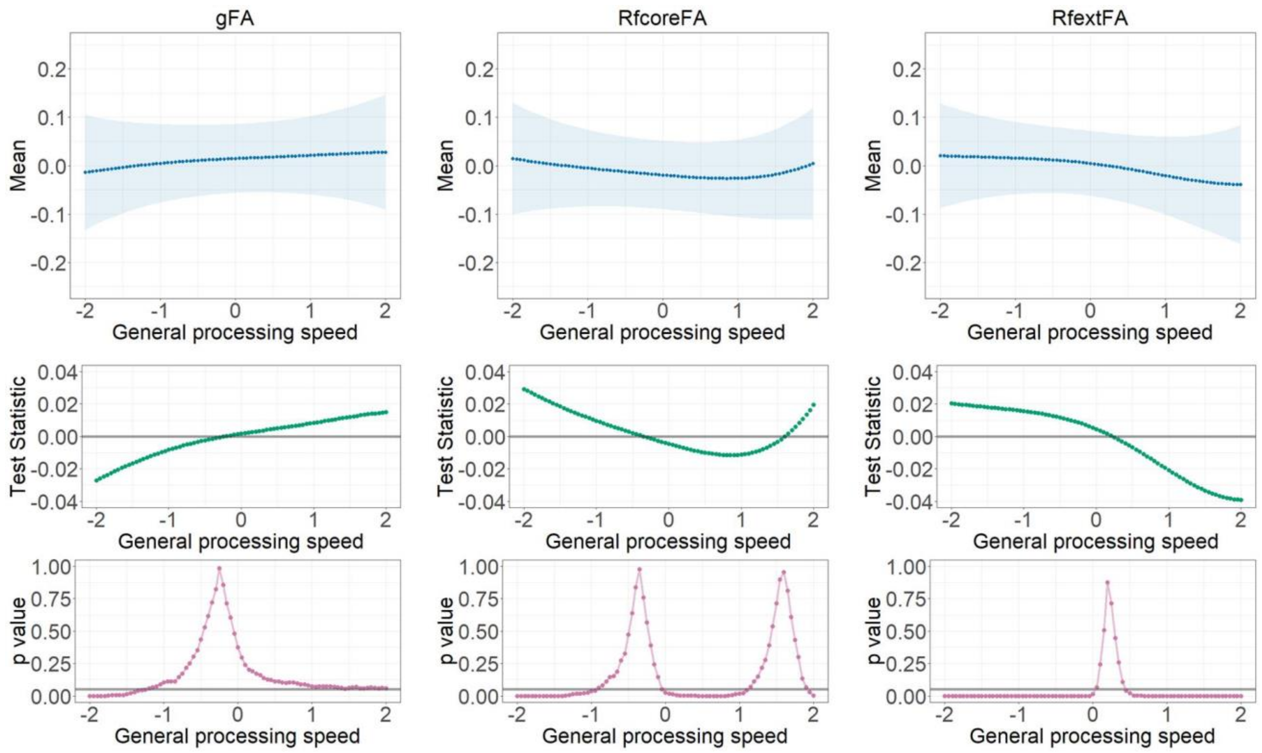

Figure S1. Parameter gradients indicating three FA factor (gFA, RfcoreFA and RfextFA) means, locally estimated across continuously assessed general processing speed ability scores (standardized factor scores) using LSEM. The upper row displays variations of locally estimated FA factor means across general processing speed ability scores – FA factor mean parameter functions. The middle row provides the course of the test statistic across general processing speed ability scores as estimated with the permutation test. The bottom row displays the pointwise *p*-value curve, with $p = .05$ displayed as a threshold (see the gray horizontal line).

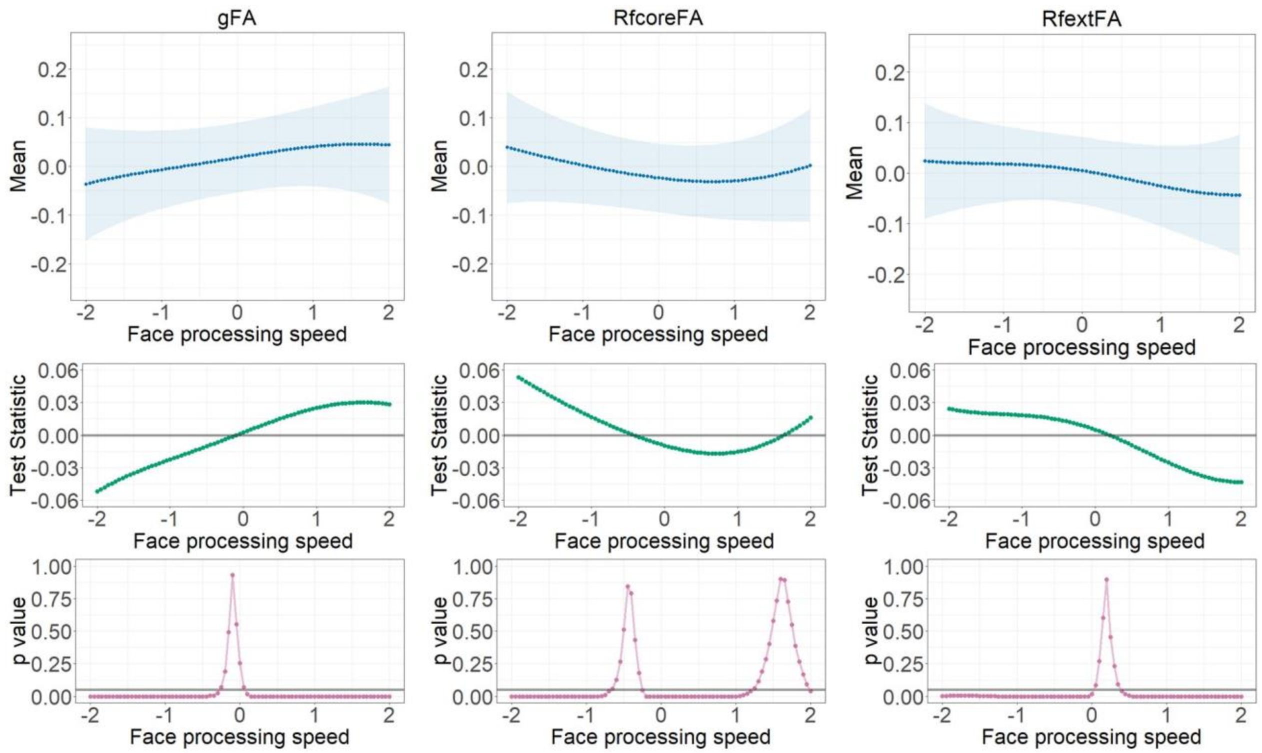

Figure S2. Parameter gradients indicating three FA factor (gFA, RfcoreFA and RfextFA) means, locally estimated across continuously assessed face processing speed ability scores (standardized factor scores) using LSEM. The upper row displays variations of locally estimated FA factor means across face processing speed ability scores – FA factor mean parameter functions. The middle row provides the course of the test statistic across face processing speed ability scores as estimated with the permutation test. The bottom row displays the pointwise *p*-value curve, with *p* = .05 displayed as a threshold (see the gray horizontal line).

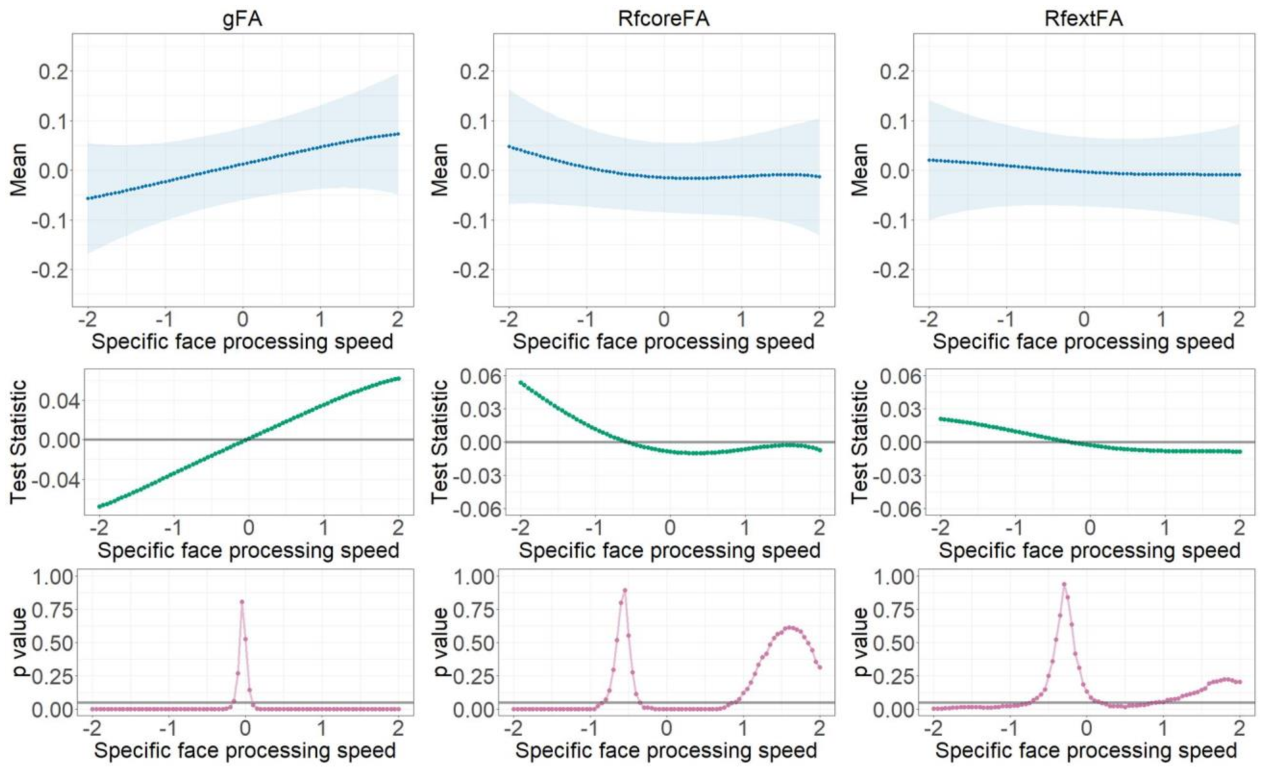

Figure S3. Parameter gradients indicating three FA factor (gFA, RfcoreFA and RfextFA) means, locally estimated across continuously assessed specific face processing speed ability scores using LSEM after partialling out the influence of the general processing speed ability. The upper row displays variations of locally estimated FA factor means across specific face processing speed ability scores – FA factor mean parameter functions. The middle row provides the course of the test statistic across specific face processing speed ability scores as estimated with the permutation test. The bottom row displays the pointwise *p*-value curve, with $p = .05$ displayed as a threshold (see the gray horizontal line).

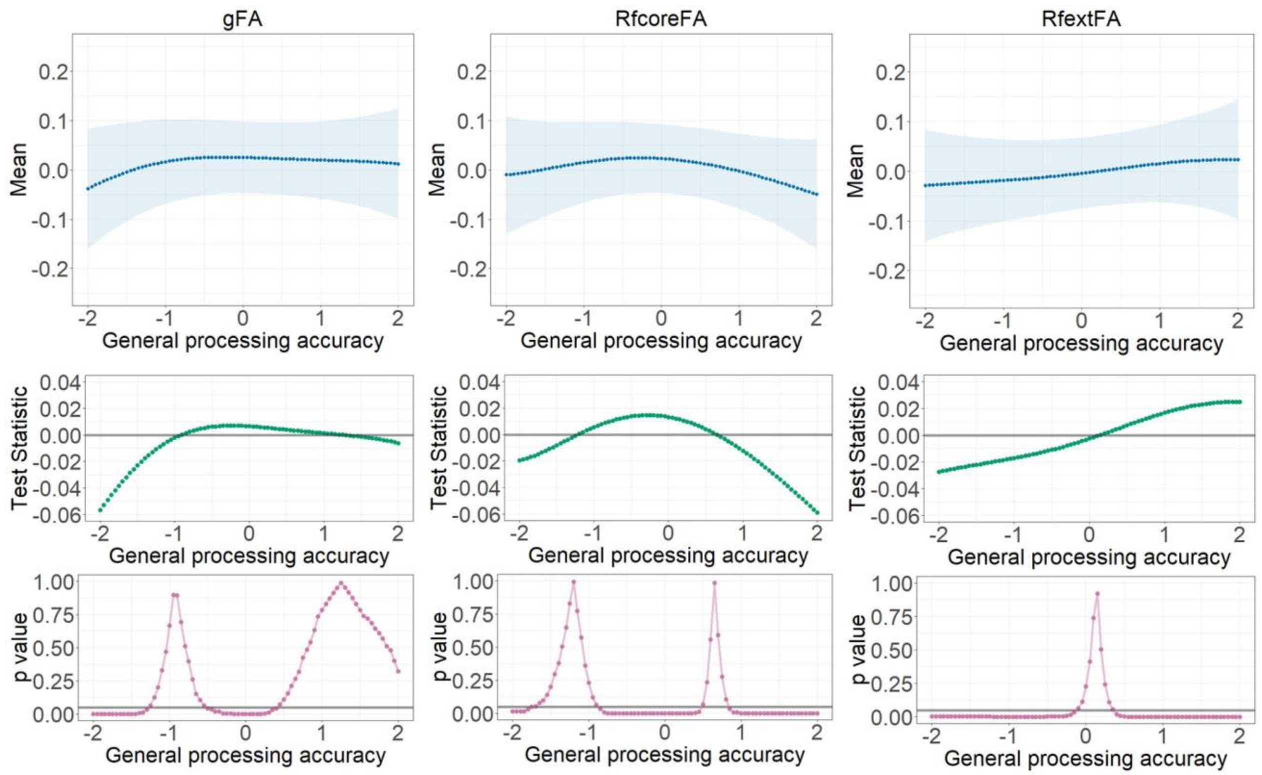

Figure S4. Parameter gradients indicating three FA factor (gFA, RfcoreFA and RfextFA) means, locally estimated across continuously assessed general processing accuracy ability scores (factor scores) using LSEM. The upper row displays variations of locally estimated FA factor means across general processing accuracy ability scores – FA factor mean parameter functions. The middle row provides the course of the test statistic across general processing accuracy ability scores as estimated with the permutation test. The bottom row displays the pointwise *p*-value curve, with $p = .05$ displayed as a threshold (see the gray horizontal line).

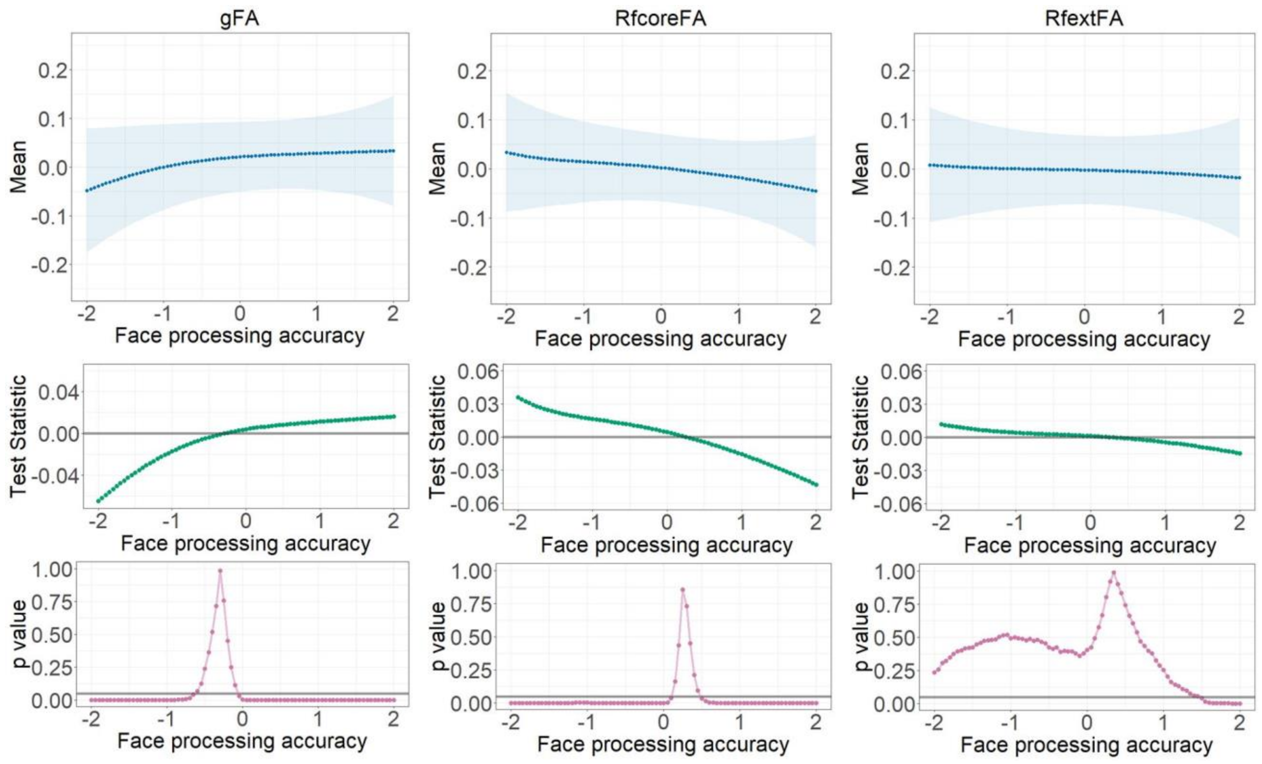

Figure S5. Parameter gradients indicating three FA factor (gFA, RfcoreFA and RfextFA) means, locally estimated across continuously assessed face processing accuracy ability scores (factor scores) using LSEM. The upper row displays variations of locally estimated FA factor means across face processing accuracy ability scores – FA factor mean parameter functions. The middle row provides the course of the test statistic across face processing accuracy ability scores as estimated with the permutation test. The bottom row displays the pointwise *p*-value curve, with $p = .05$ displayed as a threshold (see the gray horizontal line).

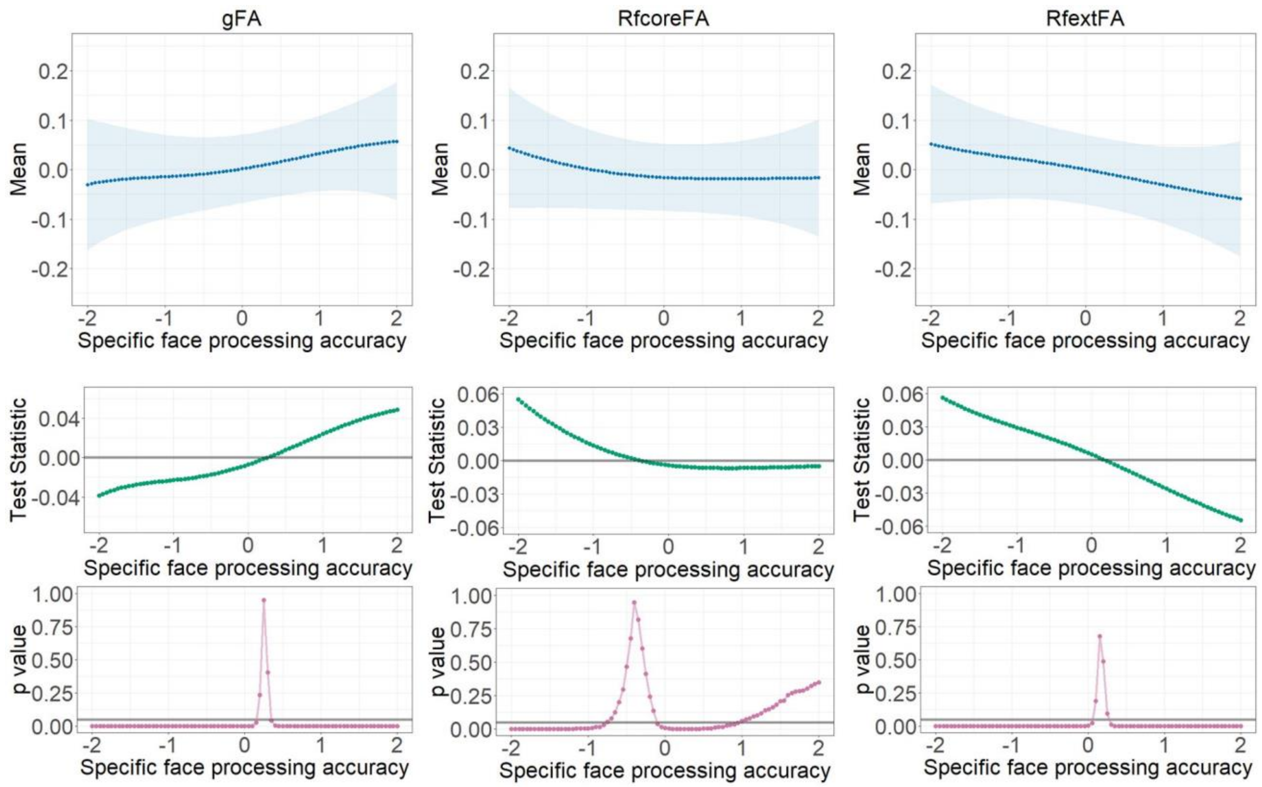

Figure S6. Parameter gradients indicating three FA factor (gFA, RfcoreFA and RfextFA) means, locally estimated across continuously assessed specific face processing accuracy ability scores (factor scores) using LSEM after partialling out the influence of the general processing speed ability. The upper row displays variations of locally estimated FA factor means across specific face processing accuracy ability scores – FA factor mean parameter functions. The middle row provides the course of the test statistic across specific face processing accuracy ability scores as estimated with the permutation test. The bottom row displays the pointwise *p*-value curve, with *p* = .05 displayed as a threshold (see the gray horizontal line).

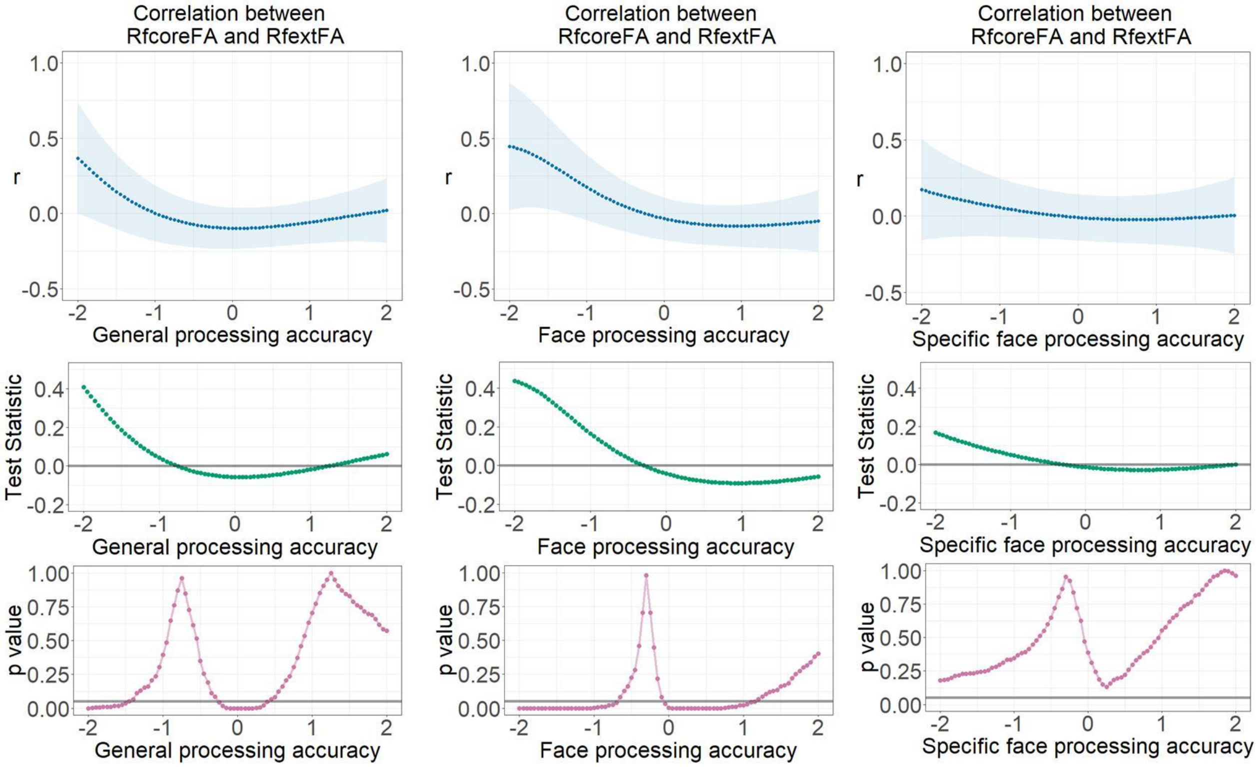

Figure S7. Parameter gradients indicating the correlation of RfcoreFA and RfextFA across continuously assessed processing accuracy ability scores using LSEM, namely 1) general processing accuracy ability, 2) face processing accuracy ability, and 3) specific face processing accuracy ability with general response accuracy controlled for. The upper row displays variations of locally estimated FA factor correlations across processing accuracy scores – FA factor correlation parameter functions. The middle row provides the course of the test statistic across response accuracy scores as estimated with the permutation test. The bottom row displays the pointwise *p*-value curve, with $p = .05$ displayed as a threshold (see the gray horizontal line). *r* – Correlation; RfcoreFA - Residual variance of the fcoreFA factor, indicating the unique variance in fcoreFA not explained by gFA; RfextFA - Residual variance of the fextFA factor, indicating the unique variance in fextFA not explained by gFA.

.

A brief description of psychometric tasks

Speed tasks

Dimensional Change ***C****ard* ***S****ort* (CS)

In each trial of the Card Sort task (Gershon et al., 2010, 2013; Weintraub et al., 2013), two target pictures were shown on the screen containing information on two content domains – shape and color. A bivalent test picture was simultaneously presented below the targets. Participants were asked to match the test picture with the targets according to shape or color.

F*lanker Inhibitory Control and Attention* ***T****ask* (FT)

In the Flanker task (Gershon et al., 2010, 2013; Weintraub et al., 2013), a sequence of arrows was presented on the screen with the central stimulus displaying the same (congruent) or opposite (incongruent) direction arrow compared with the "flankers" around. Participants were asked to focus on the central arrow and select the correct direction of the target stimulus while inhibiting their attention toward the flankers.

Pattern Comparison ***P****rocessing* ***S****peed* (PS)

In this test provided by the National Institutes of Health (NIH) toolbox, a pair of pictures were presented on the screen in each trial. Participants were asked to respond as quickly as they could by indicating whether these two pictures were the same or not.

S*ustained* ***A****ttention* (SA)

Vertical and horizontal red lines were shown on the computer screen with a flashing condition. Participants were requested to press the spacebar if they recognized a number formed by these lines in the first block and if the lines configured a letter in the second block (Gur et al., 2001, 2010; Moore et al., 2015).

***R**elational **P**rocessing* (RP)

This t-fMRI task (Smith et al., 2007) contained of a relational processing condition and a control matching condition. First, pairs of objects were shown in a row, with each pair differing either in shape or in texture. Participants decided whether the bottom pair differed in the same dimension as the top pair. In the control matching task, two objects were displayed at the top of the screen, while a reminder word appeared in the middle of the screen. Additionally, another object was presented at the bottom of the screen. Participants were requested to indicate whether the bottom stimulus belonged to the same dimension as either of the top stimuli according to the word at the center.

*Working Memory **f**acial **0**-**B**ack* (*f*0B)

In two runs of this t-fMRI procedure (Barch et al., 2013), pictures belonging to four different object categories (i.e., tools, places, faces and body parts) were presented as a 0-back and a 2-back condition. We used the former as an indicator of speed. A target cue was presented at the beginning of each 0-back run. Participants were asked to respond at each presentation of the target stimulus.

***E**motion **P**rocessing* (EP)

In two runs of this t-fMRI procedure (Hariri et al., 2002), faces with angry or fearful expressions were presented on the screen. In each trial, two facial expressions were shown at the bottom of the screen and another facial expression at the top of the screen. Participants indicated which bottom expression matched the top one.

Accuracy tasks

***O**ral **R**eading Recognition Test* (OR)

In this language task provided by the NIH toolbox (Gershon et al., 2010, 2013; Weintraub et al., 2013), participants were asked to read aloud presented words or letters from an English item bank

as accurately as possible. Scores of right or wrong were given by testers according to the performance on each trial, which are later transformed to age-adjusted individual scale scores.

V*ocabulary* ***C****omprehension* (VC)

In this task of the NIH toolbox (Gershon et al., 2010, 2013; Weintraub et al., 2013), an auditive stimulus of a single word was presented in each trial. Four pictures were simultaneously displayed on the screen. Participants were required to indicate the picture which best matched the auditive stimulus.

Rav*en Progressive Matrices* (RAV)

Fluid intelligence was measured with the Form A of the Raven's Progressive Matrices test (Bilker et al., 2012). Different patterns (i.e., 2×2, 3×3 and 1×5) of squares were presented on the screen with one piece missing. Participants were instructed to select out of five options the one that best matched the missing figures in the matric pattern.

L*ist* ***S****orting Working Memory* (LS)

In this task of the NIH toolbox (Gershon et al., 2010, 2013; Weintraub et al., 2013), a group of stimuli belonging to one dimension or two dimensions (food or/and animals) were presented both visually and orally. Participants were asked to repeat the stimuli, but reordered increasingly according to their size.

S*patial* ***O****rientation* (SO)

In each trial of the spatial orientation test (Gur et al., 2001, 2010), a movable blue line and a fixed red line were displayed together on the screen in each trial. Participants were asked to rotate the blue line clockwise or counterclockwise with the keyboard to place it parallel to the red line.

*Working Memory **f**acial **2-B**ack* (*f*2B)

In two runs of this t-fMRI procedure (Barch et al., 2013), stimuli of faces were sequentially presented on the screen – one in each trial. Participants were instructed to indicate for each face whether it was the same as the one displayed two trials back.

***F**ace **R**ecognition task* (FR)

In this task, stimuli of faces and places appeared in the working memory t-fMRI task (Barch et al., 2013) were presented again together with new faces and places. Participants were asked to indicate whether the displayed faces or places were previously shown in the t-fMRI task or not. This task was performed after the scanning session, outside the scanner.
